# Patients with low ALT levels are at increased risk for severe COVID-19

**DOI:** 10.3389/fmed.2023.1231440

**Published:** 2023-09-27

**Authors:** Dor Genzel, Lior H. Katz, Rifaat Safadi, Aliza Rozenberg, Yael Milgrom, Jeremy M. Jacobs, Asher Shafrir

**Affiliations:** ^1^Hadassah Medical School, Hebrew University of Jerusalem, Jerusalem, Israel; ^2^Gastroenterology, Hadassah Medical Center, Faculty of Medicine, Hebrew University of Jerusalem, Jerusalem, Israel; ^3^Liver Institute, Hadassah Medical Center, Faculty of Medicine, Hebrew University of Jerusalem, Jerusalem, Israel; ^4^Geriatrics and Geriatric Rehabilitation, Hadassah Medical Center, Faculty of Medicine, Hebrew University of Jerusalem, Jerusalem, Israel; ^5^Meuhedet Health Maintenance Organization, Tel Aviv, Israel

**Keywords:** alanine transaminase, sarcopenia, COVID-19, mortality, frailty

## Abstract

**Introduction:**

Frailty is a known risk factor for many diseases, including COVID-19. However, many frail patients are undiagnosed as the diagnosis can be cumbersome. Alanine transaminase (ALT) is found not only in the liver but also in the muscle tissue, and multiple studies show that frail sarcopenic patients have lower ALT. Frail patients are at increased risk for severe COVID-19. We evaluated the association between pre-infection low ALT and the risk for severe COVID-19.

**Methods:**

We collected data regarding all subjects tested for SARS-CoV-2 between 1 March 2020 and 31 December 2021 from a national state-mandatory HMO in Israel, serving more than 1.3 million patients. Clinical and laboratory data were collected, including ALT from the year prior to infection. Severe COVID-19 was defined either as death, ICU admission, or ≥10 hospitalization days. Patients with low ALT (ALT ≤ 10 IU/l) were compared with patients with normal ALT (11–40 IU/l). Patients younger than 18 years with a diagnosis of liver disease and with ALT > 40 IU/l were excluded.

**Results:**

During the study period, 58,961 patients tested positive for SARS-CoV-2. The patients in the low ALT group were younger (40.53 vs. 42.73, *p* < 0.001), less likely to be males (12.3 vs. 38.7%, *p* < 0.001), and had lower BMI (25.97 vs. 27.15, *p* < 0.001). The patients in the low ALT group had higher mortality (2.36 vs. 0.57%, *p* < 0.001), more ICU hospitalizations (0.49 vs. 0.41%, *p* = 0.47), and more prolonged hospitalizations [2.63% (95% CI 2–3.2%) vs. 0.98% (95% CI 0.86–1.1%) *p* < 0.001]. In multivariate logistic regression analyses, low ALT was associated with an increased risk of severe COVID-19, with increased mortality (OR 1.88, 95% CI 1.37–2.56) and prolonged hospitalization (OR 1.78, 95% CI 1.33–2.35).

**Conclusion:**

Low ALT level prior to infection is a significant risk factor for morbidity and mortality from COVID-19 infection. Further studies are warranted to address treatment options for this population.

## 1. Introduction

As life expectancy rises, the prevalence of frailty is expected to rise (1). Frailty is a condition defined as “a clinical state in which there is an increase in an individual's vulnerability for developing an increased dependency and/or mortality when exposed to a stressor” (2). While frailty is associated with increased all-cause mortality (1), the condition is underdiagnosed. From a public health perspective, although several measures have been proven to be useful (i.e., exercise and protein) in frail patients, (1, 3) the ability to detect patients at risk for frailty is also important.

Alanine aminotransferase (ALT) is an enzyme mainly found in the liver, kidneys, heart, brain, and skeletal muscles. ALT catalyzes the transformation of alanine to pyruvate and glutamic acid (4). Typically, elevated ALT usually indicates liver damage, and compared with other liver enzymes, ALT is considered a more specific marker of the liver (4). However, it may also indicate skeletal muscle status, as in the case where increased ALT may indicate severe rhabdomyolysis (5). In the absence of known liver damage, low ALT rates (<10–13 IU/l) are increasingly recognized as being associated with poor prognosis and increased risk of mortality (6–8), and it has been suggested that a low ALT may serve as a surrogate marker for frailty. For example, Couteur et al. studied 1,673 community-dwelling males aged 70 years or older and found patients with below-median ALT to be older and more likely to be frail (odds ratio 3.54, 95% CI 2.45–5.11) (8). Similarly, Vespasiani-Gentilucci et al. found that among 765 elderly patients (61.8% females), low ALT was associated with increased frailty phenotype (defined according to the Fried criteria) and sarcopenia (defined using the peripheral quantitative computed tomography) (9). In a prospective study from Israel among hospitalized patients, Irina et al. found a significant correlation between low ALT and both pre-frail and frailty status (classified using the FRAIL questionnaire) (10, 11). A recent study showed that patients with sarcopenia measured using the CT scan-L3 skeletal muscle index (L3SMI) had lower ALT levels (<12 IU/l) than non-sarcopenic patients (11). In addition to sarcopenia, a 5-year follow-up of a Japanese population-based cohort aged 65+ years discovered that a low ALT rate is associated with loss of independence and mortality (12).

While low ALT levels were shown in studies to be associated with increased mortality, the effect of this metabolic marker on the severity and outcomes of respiratory disease, especially COVID-19, has not been shown. According to the World Health Organization, from the beginning of the coronavirus (COVID-19) epidemic in 2019 through to the end of 2021, ~290 million people were infected, among whom more than 5 million died^1^. Multiple studies analyzed the risk factors for severe COVID-19. Among numerous variables, significant factors consistently included older age, male sex, obesity, chronic kidney diseases, diabetes, ischemic heart diseases, hypertension, asthma, and chronic obstructive pulmonary disease (COPD) (13, 14).

Frailty *per se* was not found to be associated with an elevated risk for infection with SARS-CoV-2. However, once infected with SARS-CoV-2, a frail patient has a higher risk for functional loss, increased morbidity, mortality, and increased health service utilization (15–17). Several studies showed that frailty, assessed using either the clinical frailty score (CFS) or the FRAIL scale at the time of infection immediately prior to hospitalization, is associated with an increased risk of severe COVID-19 and long-term sequelae. While these studies are important, as they were based on clinical data based on physical examination findings, numerous patients are undiagnosed (18–21). Thus, the ability of blood tests to single out frail patients is of clinical significance.

In this study, we aimed to examine the hypothesis that low baseline ALT levels prior to infection with COVID-19 were associated with an increased likelihood of severe COVID-19 infection, increased morbidity, and higher mortality.

## 2. Materials and methods

We conducted a study using the electronic medical record database of Meuhedet Health Maintenance Organization (HMO). Meuhedet HMO is the third largest state healthcare provider, with over 1.3 million patients, across all of Israel. Its database includes real-time computerized data including primary care visits, medical diagnosis, laboratory tests, hospitalizations, and prescribed medications. Data were gathered regarding all patients aged ≥ 18 years old who tested positive for COVID-19 between 1/3/20 and 31/12/21. The test was an assay from nasal and pharyngeal swabs, in accordance with World Health Organization guidelines. In cases of multiple testing, the date of the first positive test was taken to mark the onset of the disease.

Data gathered included age, sex, body mass index (BMI), and social background (based on self-definition by the patient as belonging to the general Jewish, Arab, or ultra-orthodox population). Physician-made diagnoses stored in the electronic medical records included diabetes mellitus, hypertension, ischemic heart disease, cirrhosis, chronic liver disease, dementia, history of cerebrovascular attack (CVA), COPD, asthma, and chronic renal failure. Laboratory tests for ALT levels were gathered if performed during the period spanning 30–365 days prior to COVID-19 infection. For socioeconomic status, we used the SES index which is an integral part of the HMO electronic database, automatically provided by Points Location Intelligence (https://points.co.il/). The index is determined based on residency address, according to classification by the Israel Central Bureau of Statistics. It is rated on a scale of 1–10, with 1 as the lowest. Health service utilization (length of stay in hospital, ICU admission) and mortality data were also gathered using the HMO database.

In December 2020, the first COVID-19 vaccine campaign in the world was started in Israel. The definition of vaccinated patients who were subsequently infected with SARS-CoV-2 is as follows: patients who had performed a positive SARS-CoV-2 PCR between 7 days and 5 months after receiving the second dose of the COVID-19 vaccine or 7 days after the third dose. Patients were divided into two groups: low ALT (≤10 IU/l) and normal ALT level (11–40 IU/l).

All patients included in the HMO database during the study period from 1/3/20 to 31/12/21, aged ≥ 18 years who tested positive for SARS-CoV-2 PCR were eligible for inclusion in the study. Exclusion criteria were as follows: (1) As has been done in earlier studies (22), patients with high ALT levels (>40 IU/l) were excluded since these levels represent liver disease; (2) patients with an ICD-9 diagnosis of cirrhosis or other chronic liver diseases prior to SARS-CoV-2 testing were excluded.

The measured outcomes were death, hospitalization in the ICU unit, or hospitalization of ≥10 days, all within 30 days of positive SARS-CoV-2 testing. All outcomes were mutually exclusive; that is, patients who died after a long hospitalization were not to be counted as ones who had a long hospitalization.

This research was conducted in accordance with the Declaration of Helsinki and approved by the research ethics committee and internal review board of Meuhedet HMO (02-24-08-20).

### 2.1. Statistical analysis

Descriptive statistics were used; continuous variables by mean and standard deviation and categorical by sum and relative percent. Univariate analysis for categorical variables was performed with the chi-square test and for continuous variables with Student's *t*-test. Multivariate logistic regression with odds ratio (OR) and 95% confidence interval (CI) were calculated separately for the outcomes of death, ICU hospitalization, and long hospitalization, respectively. Each model adjusted for low ALT, age, sex, socioeconomic status, sector, diabetes, hypertension, ischemic heart disease, chronic renal failure, dementia, COPD and asthma, BMI, and COVID vaccination status. In addition, a 1:1 propensity score-matched case–control analysis was performed to assess the significance of low ALT and severe COVID-19. McNemar test and Wilcoxon's singed-rank test were performed on paired data and logistic regression. To assess time to event, we constructed a Kaplan–Meier curve and calculated the difference using the log-rank test. In addition, the Cox proportional hazards model was employed to analyze the survival data in this study to adjust for multiple covariates. All tests were two-tailed, and the significance for the *p*-value was defined as <0.05%. Statistical analyses were made using R software.

## 3. Results

**Figure 1:**
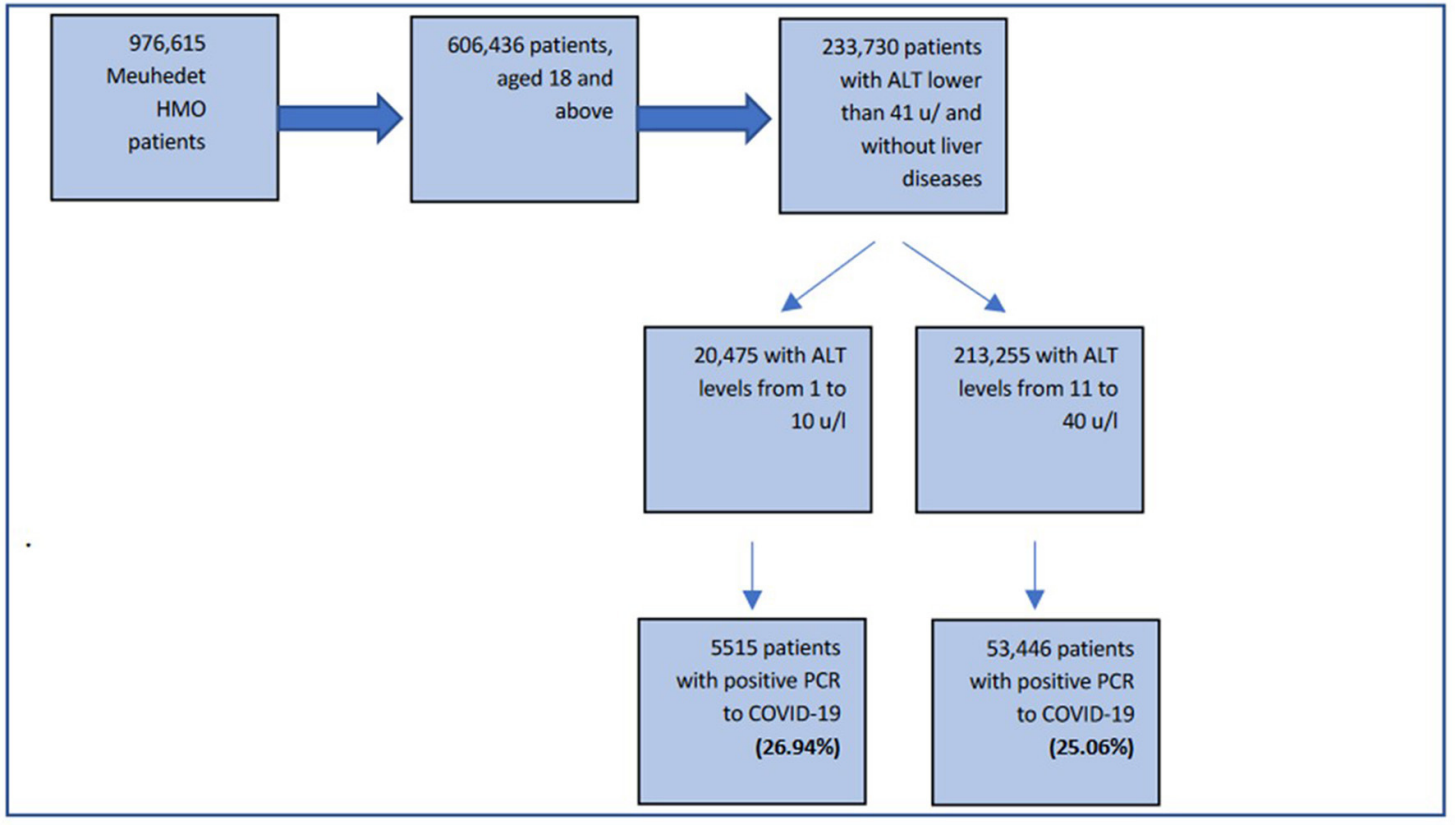
Flowchart of inclusion criteria in the study population.

During the study period (1/3/20–31/12/21), a total of 976,615 patients underwent SARS-CoV-2 PCR testing. Of these patients, we included 233,730 subjects aged ≥ 18 years, without liver diseases who had ALT levels ≤ 40 IU/l, taken between 30 and 365 days prior to testing positive for the SARS-CoV-2 PCR test. After this selection, 213,255 had normal ALT levels (11–40 IU/L) and 20,475 patients had low ALT levels (10 IU/l and below) (Figure 1). In the low ALT group, 5,515 patients (26.94% of all the low ALT group) were infected with COVID-19, and 53,446 were from the normal ALT group (25.06%, *p* < 0.001).

The patients in the low ALT group were younger (40.53 vs. 42.73, *p* < 0.001), less likely to be males (12.3 vs. 38.7%, *p* < 0.001), had lower BMI (25.97 vs. 27.15 kg/m^2^, *p* < 0.001), and were more likely to be from the Arab sector (23.0 vs. 20.3%, *p* < 0.001). Chronic renal failure (0.3 vs. 0.1%, *p* < 0.001) and dementia (1 vs. 0.2%, *p* < 0.001) were more common in the low ALT group. In addition, the patients in the low ALT group were less vaccinated against COVID-19 before the infection (4.6 vs. 5.7%, *p* = 0.002). Additional comparisons are shown in Table 1.

**Table 1:** Characteristics of the patients with positive COVID-19 PCR test, age ≥ 18 years, and without liver diseases.

	**Low ALT**	**Normal ALT**	**p-value**
	**5,515**	**53,446**	
Sex—male (%)	680 (12.3)	20,695 (38.7)	< 0.001
Age [mean (SD)]	40.53 (20.20)	42.73 (17.28)	< 0.001
Smoking (%)	321 (5.8)	4,527 (8.5)	< 0.001
Socioeconomic score (%)			< 0.001
1–3	2,155 (39.3)	19,915 (37.4)	
4–6	2,559 (46.6)	24,695 (46.3)	
7–10	775 (14.1)	8,679 (16.3)	
Sector (%)			< 0.001
Arab	1,271 (23.0)	10,848 (20.3)	
Non-Arab, non-ultra-orthodox	2,108 (38.2)	21,589 (40.4)	
Ultra-orthodox	2,131 (38.6)	20,981 (39.3)	
BMI [mean (SD)]	25.97 (5.76)	27.15 (5.73)	< 0.001
Vaccinated to COVID-19 (%)	204 (4.6)	2,377 (5.7)	0.002
ALT [mean (SD)]	8.56 (1.46)	20.79 (7.22)	< 0.001
Diabetes type 2 (%)	533 (9.7)	5,234 (9.8)	0.778
Hypertension (%)	381 (6.9)	3,658 (6.8)	0.88
Ischemic heart disease (%)	188 (3.4)	1,911 (3.6)	0.55
Congestive heart failure	181 (3.3)	831 (1.6)	< 0.001
Atrial fibrillation	177 (3.2)	1,116 (2.1)	< 0.001
Chronic renal failure (%)	14 (0.3)	38 (0.07)	< 0.001
Dementia (%)	55 (1.0)	113 (0.2)	< 0.001
COPD (%)	219 (4.0)	1,978 (3.7)	0.332
Asthma (%)	228 (4.1)	2,523 (4.7)	0.053
Stroke (%)	140 (2.5)	1,174 (2.2)	0.112

COPD, chronic obstructive pulmonary disease; ALT, alanine transaminase.

**Figure 2:**
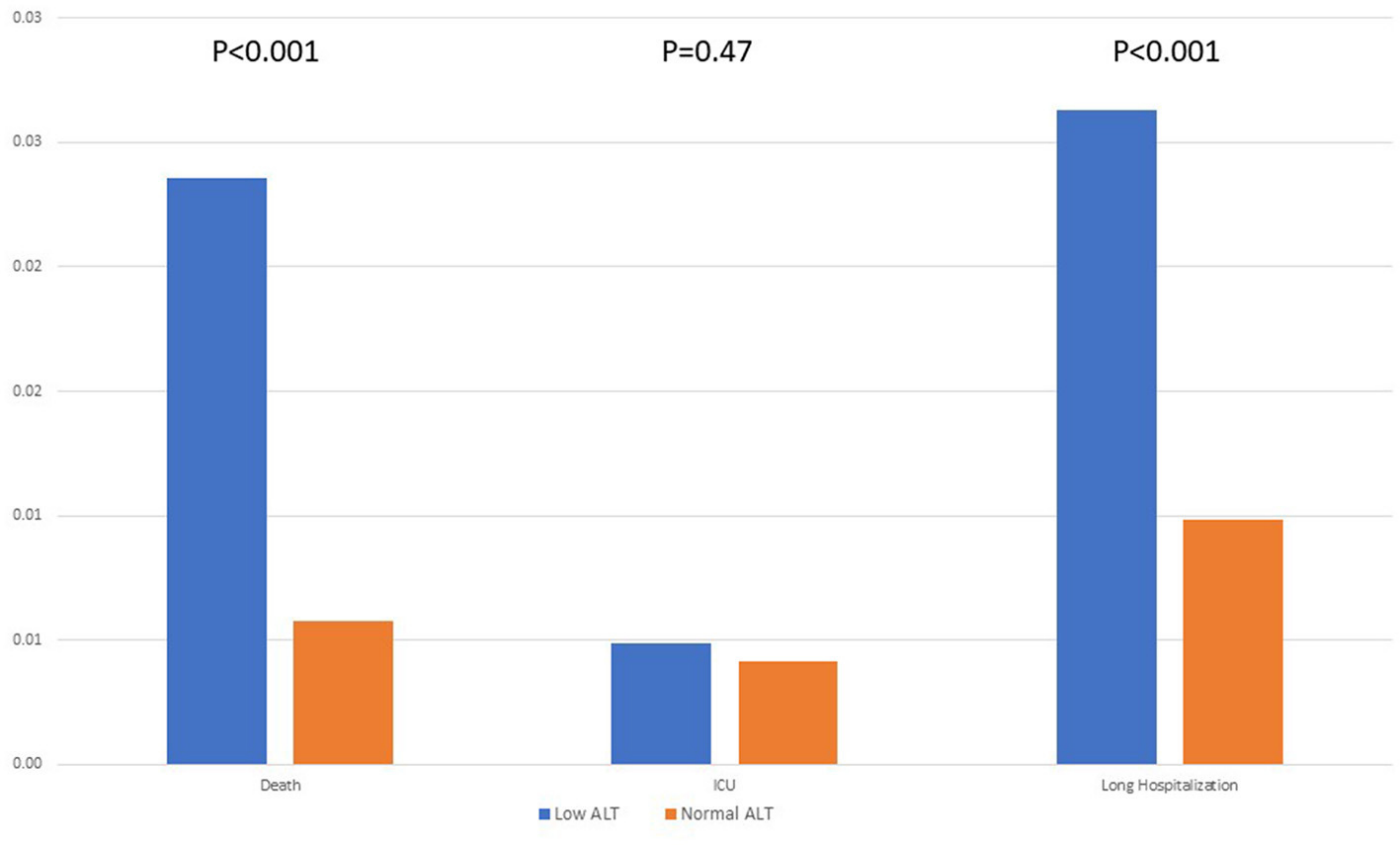
Severe COVID-19 patients divided by ALT levels.

**Figure 3:**
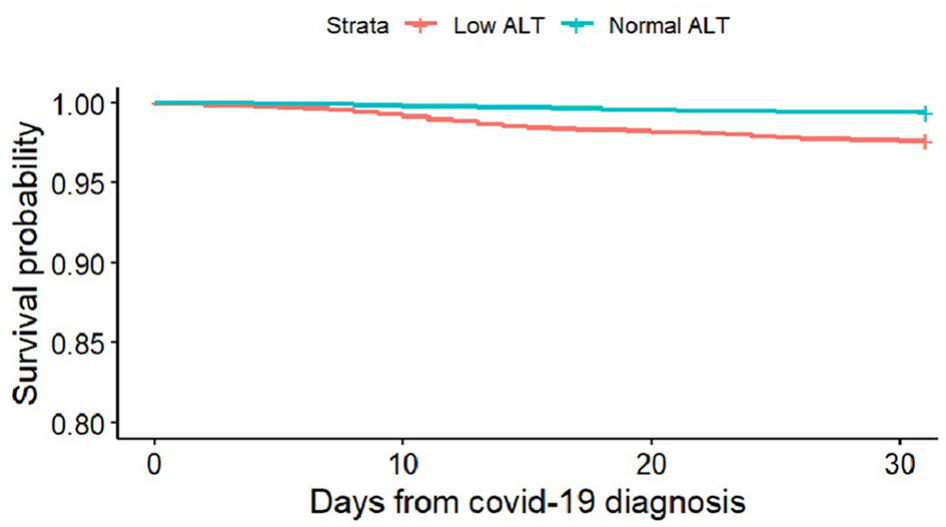
Survival analysis: days to death with COVID-19 disease, according to ALT levels.

Of all the 58,961 SARS-CoV-2-positive patients, 1,356 had severe COVID-19 infection, 437 people died, 248 were in intensive care units, and 671 were hospitalized for 10 days or more. Among the patients in the low ALT group vs. the patients in the normal ALT group, 30-day mortality was 2.36 vs. 0.57% (*p* < 0.001), and hospitalization lasting ≥ 10 days was 2.63 vs. 0.98% (*p*-value < 0.001), respectively. There was no statistically significant difference regarding ICU hospitalization (0.49 vs. 0.41%, *p* = 0.47) (Figure 2; Table 2). The difference in time to death among low and normal ALT groups was calculated using the Kaplan–Meier curve and the log-rank test. The difference was significant (*p*-value < 0.001) (Figure 3). This difference was significant when analyzing the mortality data in a Cox proportional hazards model (HR 1.71, 95% CI 1.3–2.24, *p* < 0.001).

**Table 2:** Severe COVID-19 outcomes.

	**Low ALT**	**Normal ALT**	***p*-value**
	***N* (%)**	***N* (%)**	
	**5,515**	**53,446**	
30-day mortality (%)	130 (2.36)	307 (0.57)	< 0.001
Hospitalization > 10 days (%)	145 (2.63)	526 (0.98)	< 0.001
ICU hospitalization (%)	27 (0.49)	221 (0.41)	0.47
Total events (%)	302 (5.48)	1,054 (1.97)	< 0.001

In three different multivariate logistic regressions, adjusting for low ALT, age, sex, socioeconomic status, sector, diabetes, hypertension, ischemic heart disease, chronic renal failure, dementia, COPD, asthma, BMI, COVID vaccination status, and low ALT were associated with death (OR 1.88, 95% CI 1.37–2.56, *p* < 0.001) and long hospitalization (OR 1.78, 95% CI 1.33–2.35, *p* < 0.001). In addition, in a linear regression model, increasing ALT was negatively correlated with death (coefficient estimate−7.09e-4, *p* < 0.001). Low ALT was not associated with an increased risk for hospitalization in the ICU (OR 1.2 95% CI 0.67–2.01, *p* = 0.52) (see Table 3).

**Table 3:** Risks associated with low ALT among subjects with COVID.

	**Odds ratio**	**95% CI**	***P-*value**
30-day mortality	1.88	1.37–2.56	< 0.01
ICU admission	1.2	0.67–2.01	0.52
10+ days hospitalization	1.78	1.33–2.35	< 0.01

Results of three separate logistic regression models. Each model consisted of BMI, smoking, social sector, gender, age, diabetes, hypertension, COVID-19 vaccination status, ischemic heart disease, chronic renal failure, dementia, COPD, asthma, and low ALT.

In a propensity score matched case–control analysis (*n* = 2,563 pairs), SARS-CoV-2-positive patients were matched according to gender, age, BMI, COVID-19 vaccination status, hypertension, diabetes mellitus type 2, ischemic heart disease, and socioeconomic status. Low ALT was associated with a significant risk for 30-day mortality (2.9 vs. 1.2%, *P* < 0.001), and a higher, but not significant, probability of being treated in ICU (0.6 vs. 0.5%, *p* = 0.71).

In a multivariate logistic regression, COVID-19 vaccination was associated with a lower risk of mortality (OR- 0.36, 95% CI 0.20–0.59, *p* < 0.01).

## 4. Discussion

In light of the ongoing COVID-19 pandemic, there is an urgent need to stratify the population that will be at higher risk for severe COVID-19. Multiple studies have shown that old age, male sex, hypertension, ischemic heart disease, diabetes mellitus, and history of CVA are all associated with an increased risk of severe COVID-19 (13, 14). In addition, frailty has been shown to be an additional risk factor for severe disease, death, and long hospitalization (17).

While the diagnosis of frailty as a multifaceted syndrome is clinical, with numerous tools and methods suggested to measure it, it is widely accepted that a core element of the frailty syndrome includes sarcopenia (7, 8). Sarcopenia can be measured radiologically and clinically. ALT is found throughout the body in muscle tissue, in addition to its presence in the liver, and multiple studies have confirmed that low levels of ALT are associated with sarcopenia and with a higher risk of mortality (5–11). In this study, we show an association between low ALT levels and negative outcomes for COVID-19.

The main finding in this study is that low levels of serum ALT before the disease are associated with higher mortality and longer hospitalizations from COVID-19 infection. Low ALT was a significant predictor of mortality even when controlled for other comorbidities such as age, gender, BMI, hypertension, diabetes mellitus, dementia, stroke, and pulmonary disease, with an OR = 1.88 (*p* < 0.01) in a logistic regression and a propensity score-matched cohort.

**Figure 4:**
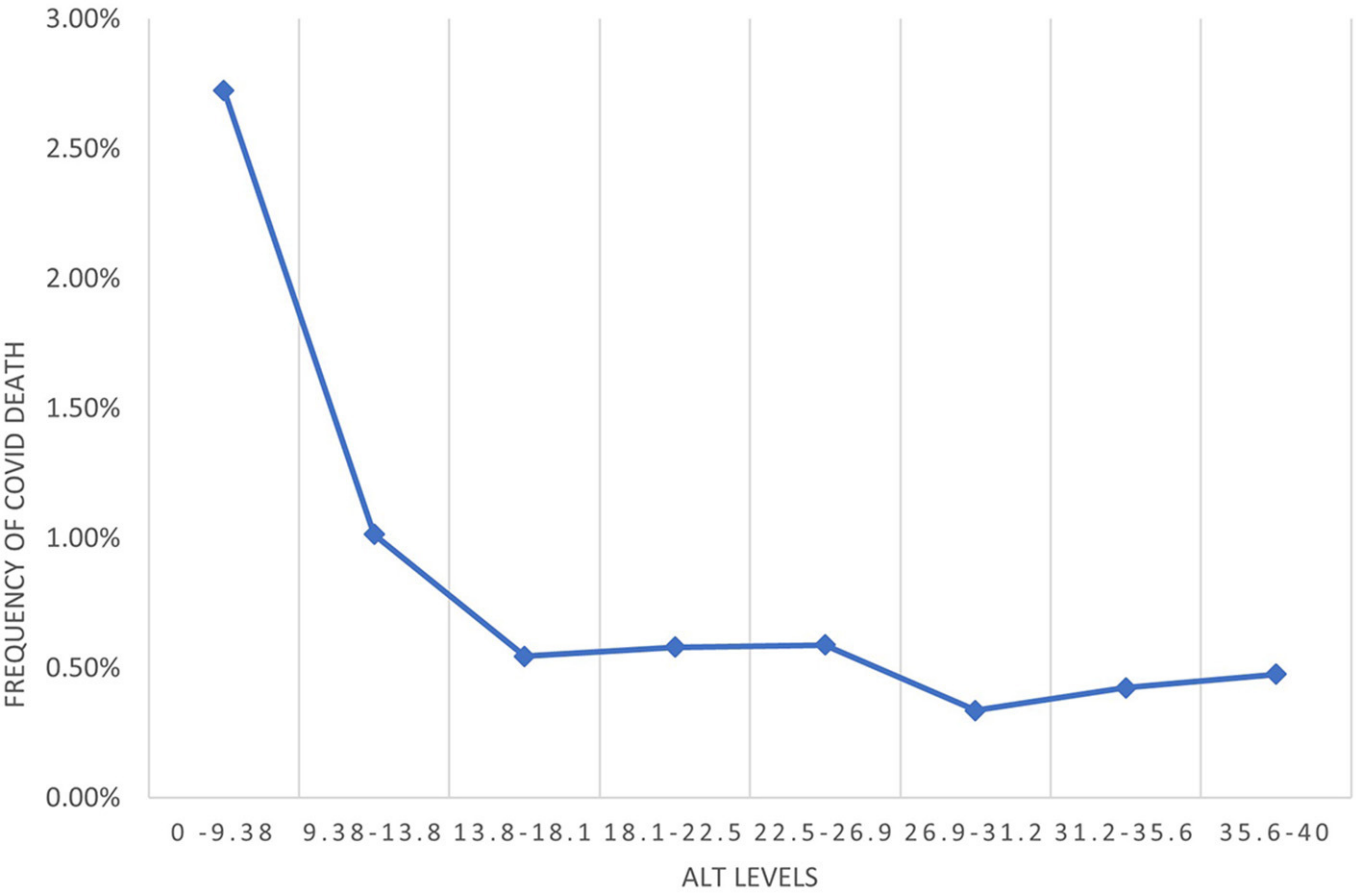
Relative incidence of death following COVID-19 infection according to ALT levels.

ALT levels < 10 U/l were considered low in our study. Other studies used a cutoff of 10–15 U/l to be considered low (7). When analyzing the incidence of death following COVID-19 infection, a significant increase was seen in patients with these low levels of ALT (Figure 4). When using a cutoff of <15 U/l, low ALT was still significantly associated with 30-day mortality when controlling for additional risk factors for severe COVID-19. This is less than the OR found when a cutoff of 10 U/l was used (Supplementary Figure S1). The best cutoff for low ALT and whether different cutoffs should be used according to sex and race should be evaluated in further studies.

Similar to other studies (19), our results showed that vaccines are associated with decreased risk for mortality (OR- 0.36, 95% CI 0.20–0.59, *p* < 0.01).

In our study, we used the ICD-10 code for chronic renal failure. As this diagnosis was not found frequently, we added an analysis where patients with serum creatinine levels > 1.5 mg/dl were considered as having chronic renal failure. High creatinine was more frequent in the low ALT group (2.4 vs. 0.9%, *p* < 0.001). When adjusting for this variable, low ALT was still significantly associated with COVID-19 death (OR 1.51, 95% CI 1.16–1.98).

Low ALT was associated with a significantly increased risk of mortality and long hospitalization. Although the risk for hospitalization in the ICU was observed to be higher in the low ALT group, nonetheless this finding did not reach statistical significance. A possible explanation could be that as the pandemic caused an overload of patients in the hospital, frail and elderly patients who were deemed to have less chance of surviving the disease were not granted an ICU bed. A similar finding was shown by Aw et al. (20).

The association of ALT and frailty is an increasingly recognized phenomenon, and it is likely that ALT serves as a surrogate marker for muscle integrity and mass. Sarcopenia is indeed a key element of frailty, and the overall integrity of the musculoskeletal system is of vital importance in the maintenance of health and functional status with advancing age. Numerous emerging models of aging indicate a shift in paradigm from a predominantly “disease-based” model to a wider inclusive concept of aging. In recent years, the World Health Organization increasingly emphasized the importance of active aging, a concept closely related to the WHO International Classification of Functioning, Disability, and Health (23, 24). Viewed from these perspectives, notions such as resilience, reserve, and intrinsic capacity are driving forces behind maintained function, health, and wellbeing during old age. Physical activity, locomotion, mobility, balance, flexibility, fatigability, nutrition, and preserved energetics are critical vectors in preventing the gradual decline and onset of frailty, which itself is viewed as a cycle of reduced activity, deteriorating intake, quantitate and qualitative muscle loss, fatigue, declining homeostasis, and energy imbalance (25, 26). Thus, the use of serum ALT as an indirect biomarker of sarcopenia and frailty is potentially an extremely useful and readily available asset in both prognostication and guidance toward more personalized care, particularly among the older patient population.

The strengths of this study are the large number of patients from a diverse population across Israel and that the study period for data collection spanned 21 months and included four pandemic waves in Israel. ALT levels were measured at least a month prior to COVID-19, and thus, there was no effect of COVID-19 itself on the ALT levels. Furthermore, as the data were collected at community clinics and not from hospitals, healthier patients were also included in the cohort.

This study has several limitations. ALT was measured only once, and only within the year prior to SARS-CoV-2 infection. Due to the retrospective nature of the study, only patients who performed an ALT test were included in the analysis, which may have introduced some degree of selection bias, as “sicker” patients might have been the ones referred to perform blood testing.

As this database is not from the hospital, there are no data regarding the disease course, such as the need for supplemental oxygen or the exact reason that caused a more difficult disease course. We used the long hospitalization as a surrogate marker for a more difficult disease trajectory. As universal medical insurance in Israel is obligatory by law, the length of stay in the hospital is not influenced by insurance-related issues and almost entirely reflects the patients' medical status. Despite these limitations, this nationwide, real-world study provides further evidence that low ALT can be used as a surrogate marker for patients at greater risk of death.

## 5. Conclusion

In conclusion, low ALT (≤10 IU/L) is a significant marker associated with severe COVID-19 for mortality and long hospitalization. Low ALT should be used as a surrogate marker of frailty and physicians and public health experts could use this biomarker for treatment and risk stratification. Further prospective studies regarding the routine use of ALT are needed.

## Author's note

This study was completed as part of the DG's MD thesis, at the Hebrew University School of Medicine, Jerusalem, Israel.

## Data availability statement

The datasets presented in this article are not readily available because there are ethical restrictions on sharing our data set because data contains potentially identifying patient information. These restrictions were imposed by the Ethics Committee of Meuhedet HMO who owns the data. Requests to access the datasets should be directed to Liron Yitzchaki, coordinator of Meuhedet Research Center, liron.y3@meuhedet.co.il.

## Ethics statement

The study was conducted in accordance with the Declaration of Helsinki and approved by the Institutional Review Board (or Ethics Committee) of Meuhedet (protocol code 02-24-08-20 given on September 02, 2020). Patient consent was waived as the data were anonymized, and no intervention occurred.

## Author contributions

DG, AS, and JJ: conceptualization, methodology, and validation. DG and AS: formal analysis, investigation, data curation, and visualization. DG: writing and original draft preparation. DG, LK, RS, YM, JJ, and AS: writing, review, and editing. JJ and AS: supervision. AR: formal analysis. All authors have read and agreed to the published version of the manuscript. All authors contributed to the article and approved the submitted version.

## Footnotes

^1^ https://covid19.who.int/
