# Supplementary figures and images for: Patients with low ALT levels are at increased risk for severe COVID-19
Source: Front Med (Lausanne). 2023 Sep 27;10:1231440. doi: 10.3389/fmed.2023.1231440 (PMC10566294; doi:10.3389/fmed.2023.1231440)

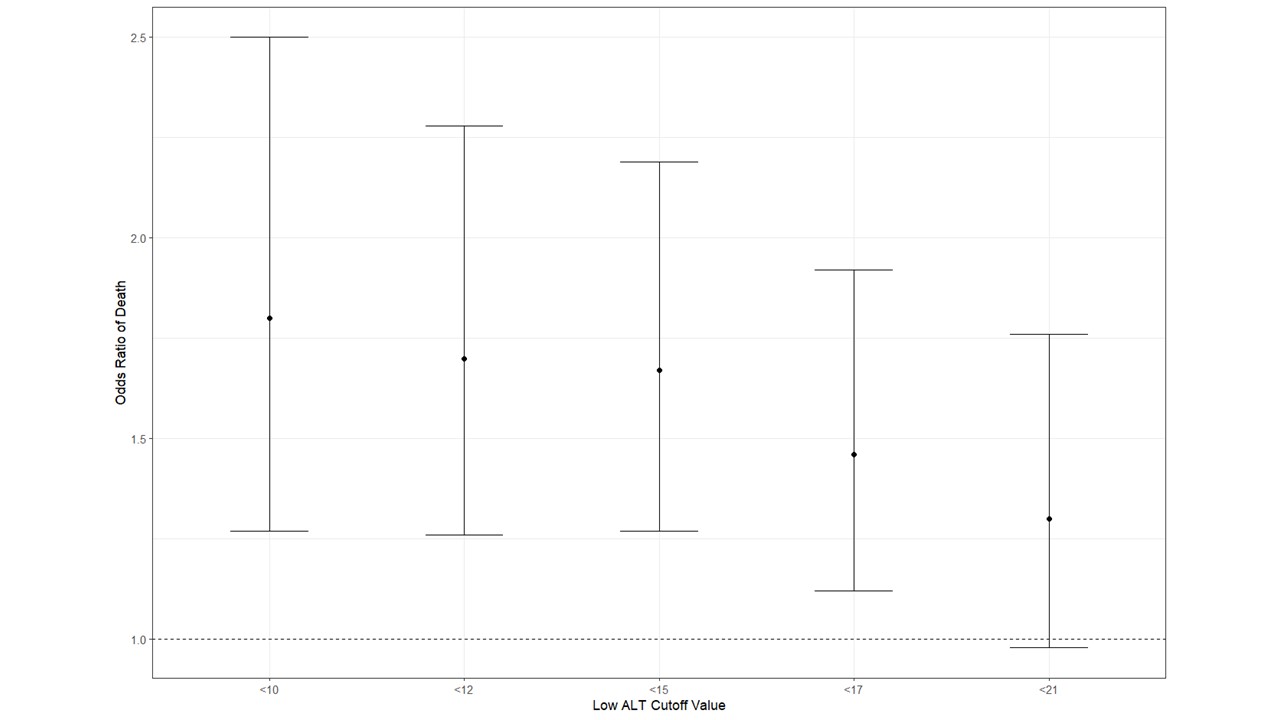

Supplement: Supplementary file 1 [file Image_1.jpg]
